# Modeling the future of tobacco control: Using SimSmoke to explore the feasibility of the tobacco endgame in Korea

**DOI:** 10.18332/tid/174127

**Published:** 2023-11-09

**Authors:** Hana Kim, Susan Park, Heewon Kang, Naeun Kang, David T. Levy, Sung-il Cho

**Affiliations:** 1 Department of Public Health Science, Graduate School of Public Health, Seoul National University, Seoul, Republic of Korea; 2 Institute of Health and Environment, Seoul National University, Seoul, Republic of Korea; 3 Lombardi Comprehensive Cancer Center, Georgetown University, Washington, United States

**Keywords:** SimSmoke, endgame, MPOWER, HP2030, modelling

## Abstract

**INTRODUCTION:**

We used a simulation model to assess the feasibility of reaching the tobacco endgame target (reducing the smoking prevalence to below 5% by 2050) and explored potential implementation strategies.

**METHODS:**

The impact of strengthened tobacco-control policies on smoking prevalence was analyzed using Korea SimSmoke, a discrete-time Markov process. We considered the effects of various scenarios from 2023 and predictions were conducted until 2050. To confirm the stability of the results, deterministic and probabilistic sensitivity analyses were carried out by increasing and decreasing parameter estimates.

**RESULTS:**

The implementation of tobacco-control policies in accordance with the WHO MPOWER (Μonitor tobacco use and prevention policies; Protect people from tobacco smoke; Offer help to quit tobacco smoking; Warn of the dangers of tobacco; Enforce bans on tobacco advertising, promotion, and sponsorship; Raise taxes on tobacco) measures were insufficient to achieve the tobacco endgame objective of 5% by 2050. The overall predicted smoking prevalence in 2050 is 4.7% if all policies are fully implemented in accordance with the FCTC guidelines together with a complete ban on the sale of cigarettes to people born after 2003 and annual 10% increases in price. Sensitivity analyses using the varying policy effect assumptions demonstrated the robustness of the simulation results.

**CONCLUSIONS:**

For a substantive reduction in smoking prevalence, it is essential to strongly implement the MPOWER strategy. Beyond this foundational step, the eradication of smoking requires a paradigm shift in the perception of conventional tobacco-control policies, including a tobacco-free generation strategy and radical increases in the price of tobacco products.

## INTRODUCTION

Globally, the smoking prevalence has significantly decreased with the implementation of tobacco-control policies^1^. Several countries, including New Zealand, Australia, England, Scotland, Ireland, Finland, and Canada, are making efforts to achieve the tobacco endgame^2^. In most countries, the endgame goal is defined as a smoking prevalence ≤5% within a timeframe between 2025 and 2040^2^. However, achieving this is hampered by the sale of highly addictive tobacco products as consumer goods^3^. To achieve the endgame, novel tobacco control policies are required^3^.

Several innovative tobacco endgame strategies have been proposed to accelerate the reduction of smoking rates^4^. For example, the tobacco-free generation (TFG) entails the prohibition of tobacco sales to certain generations^5^. Beginning in 2027, New Zealand will outlaw the sale of smoked tobacco products to the smoke-free generation born on or after 2009^6^. Annual tobacco tax increases of >10%, established as part of Australia’s and New Zealand’s tobacco regulations since 2010^7,8^, have been considered as a tobacco endgame strategy^2^.

Using simulation modeling, prior studies have predicted the effect of the TFG strategy or annual tobacco tax increases in Singapore^9,10^, the Solomon Islands^11^, and New Zealand^4,12^. The Singapore study simulated smoking prevalence and quality-adjusted life years (QALYs) based on the TFG strategy, e-cigarette use, and tax increases^9^. Cigarette eradication, tax interventions, and the TFG strategy were modeled to estimate the smoking prevalence and health-adjusted life-year (HALY) gain for the Solomon Islands^11^. The New Zealand study estimated the smoking prevalence and QALY gain under five endgame scenarios: tax increases, the TFG, outlet reduction, reduced tobacco supply, and a combination strategy^4^. These studies revealed a substantial reduction in smoking prevalence and a gain of life-years. However, they did not fully consider the effects of combinations of conventional tobacco-control policies such as designation of smoke-free areas, smoking cessation support, and warnings about the danger of tobacco. The tobacco-control policies of several countries fall short of the recommendations of the World Health Organization (WHO) Framework Convention on Tobacco Control (FCTC)^13^.

The SimSmoke model successfully predicted the smoking prevalence based on different tobacco-control policies under the FCTC guidelines in more than 27 countries and showed good results in terms of quality assessment criteria for policy simulation models, such as model input and transparency^14,15^. SimSmoke also predicted that Korea has been successful in reducing the smoking prevalence and prolonging lives^16^. Furthermore, the model has been used in research to explore the feasibility of achieving tobacco endgame goals, and SimSmoke models for the Republic of Ireland, Taiwan, and Ontario have been reported^17-19^.

In Korea, the cost attributed to cigarette smoking exceeded KRW 12 trillion (US$ 9 billion) in 2019, including more than KRW 4 trillion (US$ 3 billion) in medical costs^20^. As of 2021, the adult (age ≥19 years) male smoking prevalence was 31.3%, which is higher than the average of countries in the Organization for Economic Cooperation and Development (OECD)^21^. To ameliorate the devastating effects of smoking, the Korean government established the Health Plan (HP) 2030, which aims to reduce the smoking rate among males by 25% by 2030^22^. In the 2019 National Tobacco Control Plan, the Korean government alluded to a tobacco endgame^23^. However, the strategies to strengthen tobacco-control policies were not clearly delineated, and the proclamation of the tobacco endgame lacked a target date and prevalence^23^.

We investigated whether the tobacco endgame is achievable by 2050 in Korea with various traditional tobacco-control policies together with the TFG strategy and annual increases in the price of cigarettes using the Korea SimSmoke model. In addition, we explored strategies for achieving the goal of the HP in Korea.

## METHODS

### Korea SimSmoke model and policy enforcement scenarios

*Model description*

In the Korea SimSmoke model, a discrete-time Markov process was used to forecast the future population and number of people who smoke, as reported previously^16^. The SimSmoke model was programmed with Excel software^24^. We focused exclusively on the use of conventional cigarettes and did not take into account other tobacco products such as electronic cigarettes (ECs) and heated tobacco products (HTPs).

The future population by age and sex was predicted based on the 2001 fertility rates, 2005 mortality rates, and the 1995 Census, all from the Korea National Statistical Office. The future number of people who smoke by age and sex was calculated based on the smoking initiation rate, quit rate, and smoking relapse rate, which were obtained from the 1995 and 1998 Korea National Health and Nutrition Examination Surveys (KNHANES). To assess the effects of tobacco-control policies on smoking prevalence, a policy module was included. The policy-module parameters were obtained from previous studies and expert interviews in Korea, because of the lack of studies of direct effects of the seven tobacco-control policies, the exception being pricing^16^. Table 1 lists the effect sizes of SimSmoke policy modules reflected by MPOWER (M, monitor tobacco use and prevention policies; P, protect people from tobacco smoke; O, offer help to quit tobacco smoking; W, warn of the dangers of tobacco; E, enforce bans on tobacco advertising, promotion, and sponsorship; R, raise taxes on tobacco), which is a set of demand-reduction measures recommended by WHO as part of FCTC.

**Table 1:** SimSmoke model policy effect size by WHO MPOWER strategy

**MPOWER**	**P**rotect people from tobacco smoke	**O**ffer help to quit tobacco use	**W**arn about the dangers of tobacco	**E**nforce bans on tobacco advertising, promotion and sponsorship	**R**aise taxes on tobacco
**FCTC Article**	(8) Protection from exposure to tobacco smoke	(14) Demand reduction measures concerning tobacco dependence and cessation	(11) Packaging and labelling of tobacco products	(13) Tobacco advertising promotion and sponsorship	(6) Price and tax measures to reduce the demand for tobacco
**SimSmoke Model**	Clean air laws Smoking ban in indoor workplaces and restaurants	Smoking cessation treatment Financial coverage of pharmaceutical and behavioral treatment, brief intervention, and quitline	Health warnings High: labels are large, bold, and graphic, and cover ≥30% of pack Low: labels are not bold and graphic, and cover <30% of package	Advertising ban Comprehensive: ban is applied to television, radio, print, billboard, in-store displays and sponsorships Complete: ban is applied to all media, television, radio, print, billboard Partial: ban is applied to some of television, radio, print, billboard	Price policy Tobacco price and tax
SimSmoke effect size*	Indoor workplaces: Complete 6% Partial 2% Restaurants: Complete 1% Partial 0.5% Bars, etc.: 0.5%	For high-intensity reduced smoking rate by 2.6% and increased smoking cessation rate by 50%	High (2% smoking prevalence, 2% initiation rate, 4% cessation rate) Low (1% smoking prevalence, 1% initiation rate, 2% cessation rate) *Enforcement, Publicity 1%	Comprehensive ban (6% smoking prevalence, 8% initiation rate, 3% cessation rate) Complete ban (4% smoking prevalence, 6% initiation rate, 2% cessation rate) Partial restriction (1% smoking prevalence, 1% initiation rate) *Enforcement, Publicity 1%	Price elasticity: Age 15–17 years, -0.4 Age 18–24 years, -0.3 Age 25–34 years, -0.2 Age ≥35 years, -0.1

* If there is no detailed explanation, the effect size is applied equally to the reduction in smoking rate, smoking initiation rate, and increase in smoking cessation rate of all ages and genders.

*Policy enforcement scenarios*

The model tracked smoking prevalence from 1995 to 2022 and forecast the smoking prevalence for 30 years from 2023. The model predicted the smoking prevalence based on the status quo scenario, assuming that the current policy status continues. It was also predicted according to the tobacco-control policy strengthening scenario, which assumes implementation of non-price policies (POWE of MPOWER), a price policy (R of MPOWER), and a TFG strategy individually or in combination. The non-price policies were strengthened from 2023. Cigarette price policy was applied based on a scenario in which the cigarette price increased to KRW 8000 (US$ 7.36) in the first year, similar to the OECD average cigarette price, and increased by 10% annually thereafter. The TFG strategy assumed a lifetime ban on the sale of cigarettes to people born after 2003, based on the assumption that smoking would be illegal for those aged ≤20 years in 2023. Accordingly, the annual smoking initiation rate was set at 0% from 2023.

### Evaluation of Korea’s implementation of tobacco-control policies

*Protect people from tobacco smoke (P)*

The clean air policy module examined the effects of laws related to workplaces, restaurants, and other public places^16^. The Korean government gradually expanded smoke-free areas based on the National Health Promotion Act in 1995 and designated most public places, including all restaurants, as smoke-free areas in 2015^25^. Although there are penalties for non-compliance, the laws have not been enforced on a large scale. Therefore, we consider the clean-air policy to be a partial (50%) implementation.

*Offer help to quit tobacco use (O)*

The cessation treatment policy in SimSmoke^26^ considers the effects of: 1) mandated brief interventions provided by a physician, 2) complete financial coverage of cessation treatments, and 3) a telephone quitline service. The Korean government has operated smoking cessation clinics in public health centers, a proactive quitline service, and has provided financial coverage for cessation treatment in designated clinics^27^. However, brief intervention programs have not yet been implemented and physicians do not generally provide these interventions, and financial coverage was restricted to designated clinics.

*Warn of the dangers of tobacco (W)*

In SimSmoke, health warnings are defined as weak or strong. A warning that is bold and graphic and covers at least one-quarter of the front of the package is defined as strong. Beginning in 2016, graphical health warnings on the front and back of the package covering >30% of the surface area were required. Therefore, the health-warning parameter was set as strong in SimSmoke.

*Enforce bans on tobacco advertising, promotion, and sponsorship (E)*

In the model, a total ban was applied to all media, whereas a partial ban was applied to television and some other media. The model also incorporated enforcement and publicity. The bans on tobacco advertising, promotion, and sponsorship were assessed as low because some tobacco advertisements were allowed, and tobacco promotions and tobacco companies’ social contribution activities were not prohibited^27^. The model characterizes this as a partial advertising ban.

*Raise taxes on tobacco (R)*

The SimSmoke model calculates the effect of price changes on smoking prevalence by applying an equation that depends on the elasticity of demand. We set the average retail price of cigarettes as KRW 4500 per pack and taxes accounted for 73.9% of the retail price from 2015 in the SimSmoke model^28^. Past price changes were deflated based on the consumer price index. For price changes after 2023, the inflation rate was assumed to be 3%.

### Model validation and sensitivity analyses

To validate the predictions, data from the nationally representative KNHANES, Social Statistics Survey, and Community Health Survey (CHS) were compared with the smoking prevalence predicted by SimSmoke. For comparison, smoking prevalences were obtained from the 1995, 1998, 2001, 2005, and 2007 to 2020 KNHANES^29^. Using Social Statistics Survey, smoking prevalences were compared in 1995, 1999, 2003, and from 2006 to 2018 on a 2-year basis^30^. We also used the annual CHS reports on smoking prevalence from 2008 to 2020^31^. Because the female smoking prevalence in the CHS is not publicly available, it was omitted.

To examine the effects of future policies, two sensitivity analyses were conducted to confirm output stability according to changes in model parameter-effect sizes, intended as an element of the assessment of the quality of the tobacco-control policy simulation model^15^. One-way sensitivity analyses were performed to evaluate the effects of varying parameters of each POWER policy. Parameter variation of ± 50% was assumed based on the endgame achievement scenario^32^. A probabilistic sensitivity analysis was conducted to evaluate the impact of uncertainty in the parameter estimation (Supplementary file Section 1). The values of the parameters were randomly selected (1000 samples) from a beta distribution for the policy parameters using bootstrapping. Next, we calculated 95% confidence intervals based on bias-corrected percentile method adjustments for bootstrap estimation^33^.

## RESULTS

### Model validation

The smoking prevalence by gender, forecasted by the Korea SimSmoke model, was compared with the survey data (Supplementary file Section 2). For males, there was a small deviation between the predicted and surveyed prevalence values, and the difference decreased over time, suggesting the validity of the model. In contrast, the smoking prevalence predicted by the SimSmoke model was less valid for females, as indicated by the marked difference with the survey data. The SimSmoke model over- and under-predicted, respectively, the decline relative to the KNHANES and Social Statistics Survey data.

### Prediction of smoking prevalence by scenario

Seven scenarios for strengthening tobacco-control policies were considered to reach the target of a <5% smoking prevalence: 1) a status quo scenario in which the 2022 tobacco-control policy level would be maintained; 2) a POWE strategy involving full-strength non-price policies, such as tobacco-free areas in all indoor workplaces and restaurants, smoking cessation treatments with mandatory brief interventions, and bans on all tobacco advertisements, promotion and sponsorships; 3) cigarette price increase to KRW 8000 per pack (denoted as R8000) combined with a POWE strategy; 4) annual 10% cigarette price increase with an initial increase to KRW 8000 per pack (denoted as R8000 + R10%) in combination with a POWE strategy; 5) TFG strategy, 0% smoking initiation in the generation born in 2003, together with a POWE strategy; 6) TFG, POWE, and R8000; and 7) TFG, POWE, and R8000 + R10%.

The forecast smoking prevalence among males is shown in Table 2. The predicted smoking prevalence among males in 2030 did not decrease to <25% when only non-price policies were fully implemented, whereas it was predicted to be <25% in the scenarios including non-price policies with a cigarette price of KRW 8000 or implementation of the TFG strategy in 2023. Implementation of all non-price policies together with the TFG and a 10% annual increase in price was predicted to result in a smoking prevalence among males in 2050 of 8.5%.

**Table 2:** Predicted smoking prevalence (%) in males under strengthened policy scenarios from 2022 to 2050 in Korea

*Scenario*	*2022*	*2025*	*2030*	*2035*	*2040*	*2045*	*2050*
Status quo^a^	35.3	34.0	32.2	30.7	29.4	28.3	27.6
POWE^b^	35.3	29.2	27.0	25.1	23.5	22.2	21.1
POWE^b^ + R8000^c^	35.3	27.0	24.8	22.9	21.2	19.9	18.7
POWE^b^ + R8000^c^ + R10%^d^	35.3	26.3	22.7	19.7	17.2	15.1	13.4
POWE^b^ + TFG^e^	35.3	28.8	24.9	21.2	17.9	15.0	12.3
POWE^b^ + TFG^e^ + R8000^c^	35.3	26.7	22.9	19.4	16.4	13.6	11.1
POWE^b^ + TFG^e^ + R8000^c^ + R10%^d^	35.3	26.0	21.1	17.0	13.6	10.8	8.5

Apply each strengthened policy scenario from 2023.

a Maintain the current tobacco control policy.

b POWE: P, protect people from tobacco smoke. O, offer help to quit tobacco smoking. W, warn of the dangers of tobacco. E, enforce bans on tobacco advertising, promotion, and sponsorship.

c Cigarette price increase to KRW 8000.

d Annual 10% cigarette price increase from KRW 8000.

e TFG: Tobacco-Free Generation. We assumed that persons born after 2003 would not start smoking.

As shown in Table 3, the predicted smoking prevalence among females was 1.2% in 2050 with implementation of all the non-price policies, a 10% annual price increase annually, an initial increase in cigarette price to KRW 8000, and the TFG strategy.

**Table 3:** Predicted smoking prevalence (%) in females under strengthened policy scenarios from 2022 to 2050 in Korea

*Scenario*	*2022*	*2025*	*2030*	*2035*	*2040*	*2045*	*2050*
Status quo^a^	3.6	3.5	3.3	3.3	3.2	3.1	3.1
POWE^b^	3.6	3.0	2.8	2.7	2.6	2.5	2.4
POWE^b^ + R8000^c^	3.6	2.8	2.6	2.5	2.4	2.3	2.2
POWE^b^ + R8000^c^ + R10%^d^	3.6	2.7	2.4	2.1	1.9	1.7	1.6
POWE^b^ + TFG^e^	3.6	2.9	2.6	2.3	2.1	1.9	1.7
POWE^b^ + TFG^e^ + R8000^c^	3.6	2.7	2.4	2.2	1.9	1.7	1.5
POWE^b^ + TFG^e^ + R8000^c^ + R10%^d^	3.6	2.7	2.2	1.9	1.6	1.4	1.2

Apply each strengthened policy scenario from 2023.

a Maintain the current tobacco control policy.

b POWE: P, protect people from tobacco smoke. O, offer help to quit tobacco smoking. W, warn of the dangers of tobacco. E, enforce bans on tobacco advertising, promotion, and sponsorship.

c Cigarette price increase to KRW 8000.

d Annual 10% cigarette price increase from KRW 8000.

e TFG: Tobacco-Free Generation. We assumed that persons born after 2003 would not start smoking.

**Figure 1:**
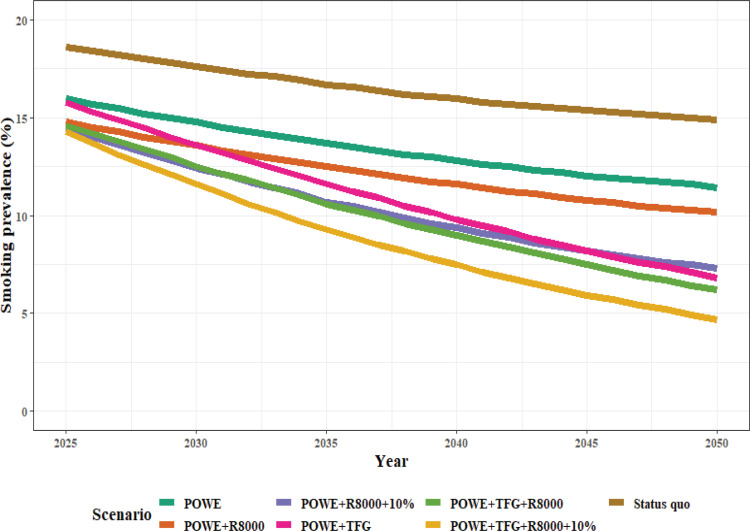
Predicting smoking prevalence by policy reinforcement scenario from 2025 to 2050 in Korea

These results suggest that the implementation of a TFG strategy in conjunction with POWE, R8000, and R10% is crucial to achieve the tobacco endgame, i.e. a smoking prevalence of <5% for both genders in 2050 (Figure 1).

### Sensitivity analysis

We conducted one-way sensitivity analysis of the SimSmoke projection from 2030 to 2050 with full implementation of non-price policies, TFG, R8000, and R10%. As shown in Table 4, the policy parameters were adjusted from -50% to 50% of the respective effect sizes^32^. The predicted smoking prevalence was most affected by the change in the price policy parameter (R). The difference between the predicted smoking prevalence values was approximately 2%p when the R parameter value was altered by 50%. The smoking cessation service (O) parameter had the least impact on smoking prevalence. The difference in predicted smoking prevalence was within 0.01%p when the O parameter was altered by 50%.

**Table 4:** Sensitivity analysis of the SimSmoke model (%) in 2030 and 2050 in Korea

*MPOWER*	*Range*	*Males*		*Females*		*Total*	
		*2030*	*2050*	*2030*	*2050*	*2030*	*2050*
**P**	+50	19.8	7.8	2.1	1.1	10.8	4.3
	+25	20.4	8.1	2.2	1.1	11.2	4.5
	0	21.1	8.5	2.2	1.2	11.6	4.7
	-25	21.8	8.8	2.3	1.2	11.9	4.9
	-50	22.5	9.2	2.4	1.2	12.3	5.1
**O**	+50	21.1	8.5	2.2	1.2	11.6	4.7
	+25	21.1	8.5	2.2	1.2	11.6	4.7
	0	21.1	8.5	2.2	1.2	11.6	4.7
	-25	21.1	8.5	2.2	1.2	11.6	4.7
	-50	21.1	8.5	2.2	1.2	11.6	4.7
**W**	+50	20.7	8.2	2.2	1.1	11.3	4.6
	+25	20.9	8.4	2.2	1.1	11.4	4.6
	0	21.1	8.5	2.2	1.2	11.6	4.7
	-25	21.3	8.6	2.3	1.2	11.7	4.7
	-50	21.5	8.7	2.3	1.2	11.8	4.8
**E**	+50	20.2	8.0	2.1	1.1	11.0	4.4
	+25	20.6	8.2	2.2	1.1	11.3	4.6
	0	21.1	8.5	2.2	1.2	11.6	4.7
	-25	21.6	8.7	2.3	1.2	11.8	4.8
	-50	22.1	8.9	2.3	1.2	12.1	4.9
**R**	+50	17.7	6.3	1.9	0.9	9.7	3.5
	+25	19.3	7.3	2.1	1.0	10.6	4.1
	0	21.1	8.5	2.2	1.2	11.6	4.7
	-25	23.1	9.8	2.4	1.3	12.6	5.4
	-50	25.2	11.4	2.7	1.5	13.8	6.3

P: protect people from tobacco smoke. O: offer help to quit tobacco smoking. W: warn of the dangers of tobacco. E: enforce bans on tobacco advertising promotion and sponsorship. R: raise taxes on tobacco. The sensitivity analysis was conducted under the combined POWE, TFG, R8000, and R10% scenario.

## DISCUSSION

An HP2030 target (25% of the male smoking prevalence) and a 2050 tobacco endgame goal (5% of the total smoking prevalence) could be achieved in Korea by implementing a comprehensive POWER policy and introducing innovative strategies. Specifically, the smoking prevalence among males was estimated to be 24.8% in 2030 if the cigarette price were to be increased to 8000 KRW, together with a high-intensity non-price policy beginning in 2023. Similarly, a ban of tobacco sales to the generation born in 2003 without a cigarette price increase would result in a male smoking prevalence in 2030 of 24.9%. The tobacco endgame could be achieved by high-intensity non-price policies (POWE), the TFG strategy, and a cigarette price increase to KRW 8000 with a 10% increase annually thereafter.

Our findings suggest the need to increase the tobacco price in Korea and other countries where the cigarette price is lower than the OECD average. If only high-intensity non-price policies were implemented, the male smoking prevalence in 2030 decreased by 5.2%p compared to the *status quo*; by contrast, if the cigarette price was increased to 8000 KRW in 2023 with high-intensity non-price policies, the male smoking prevalence in 2030 was predicted to decrease by 7.4%p compared to the status quo. Increasing the cigarette price without a discount was projected to significantly reduce the smoking prevalence in the United States^34^. Also, the Canada SimSmoke model showed that strengthening of a price policy leads to major reductions in smoking prevalence^19^.

If the cigarette price is increased by 10% annually after 2023, Korea SimSmoke predicted that the male smoking prevalence in 2030 would decrease by 9.5%p compared to the status quo. A regular tobacco tax increase is important to achieve the endgame target of <5% smoking prevalence^35^. In a modelling study conducted in New Zealand, a 10% annual increase in tobacco tax with a non-price policy significantly decreased the smoking prevalence^35^. Regular tax increases could outweigh the short-term effects of increased illicit trade, although extremely high cigarette prices are likely to lead to use of alternative tobacco products and/or bolster the illegal tobacco trade^35^.

Korea has maintained the current cigarette price of KRW 4500 (US$ 3.79), less than the OECD average of USD 6 since an increase in 2015 from KRW 2500 (US$ 2.2)^36^. The proportion of smoking households and the average monthly tobacco consumption of smoking households decreased after the price increase^37^. Indeed, the decline was induced after the short-term effect faded following implementation of the price policy^37^. In addition, Korea’s tobacco excise tax is 73.9%, which is close to that (75% of the retail price) recommended by the FCTC, but is still insufficient^28^. Therefore, increasing the cigarette price to the OECD average is required to achieve the HP2030 target and a smoking prevalence of <5%.

We devised a scenario in which tobacco sales are prohibited to people born after 2003 beginning in 2023 in Korea. In fact, beginning in 2027 New Zealand plans to prohibit tobacco sales to anyone born on or after 2009^6^. Moreover, several countries, including Denmark, Spain, Ireland, Singapore, and Malaysia, are considering adopting the TFG^6,38^. Our findings indicate that the male smoking prevalence will decrease by 8.8%p in 2050 if non-price policies are implemented in conjunction with the TFG strategy in 2023, compared to implementation of only high-intensity non-price policies. These results are in line with those of previous modeling studies. Singh et al.^11^ reported that residents of the Solomon Islands would be expected to gain 798 HALYs per 1000 people if smoking was restricted to those ≤20 years of age from 2016^11^. Furthermore, the tobacco endgame goal was predicted to be attained by the TFG alone, which was expected to be more effective than gradually raising the minimum legal age of smoking to 25 years^10^. However, it will take a long time to lower smoking prevalence and reap health gains because current smokers are unaffected by the TFG^10,12^. Therefore, we suggest introducing the TFG together with aggressive tax increases and implementation of MPOWER policies to end the tobacco epidemic.

In this study, full implementation of the POWER policies, combined with the introduction of endgame strategies such as the TFG strategy and a consistent 10% annual increase in cigarette prices, was projected to decrease the smoking prevalence to 5% by 2050. Based on FCTC evaluation of the targets of MPOWER, the Korean government should strengthen smoking bans (P) and advertising bans (E)^13^. The experts jointly recommended implementation of MPOWER as a roadmap to achieve the tobacco endgame^39^. Therefore, for a rapid decrease in smoking prevalence, it is essential to implement MPOWER concurrently with a variety of endgame strategies.

The female smoking prevalence predicted by SimSmoke differed markedly from the survey data. The difference in the female smoking prevalence between that in KNHANES and predicted by SimSmoke each year ranged from 0.42%p to 3.78%p. The difference could be caused by survey inaccuracy^40^. The number of females who reported themselves to be current smokers in the KNHANES was less than half of the number identified based on the urine concentration of cotinine^40^. This discrepancy could result from social repression of smoking and reporting among Asian females, as well as gender differences in smoking patterns^40^.

### Strengths and limitations

The strength of this study was in the simulation of the effects of conventional tobacco-control policies combined with a variety of endgame strategies, such as the TFG strategy and a consistent 10% annual increase in cigarette prices. However, this study had several limitations. First, we examined only the impact of tobacco-control policies on conventional cigarettes. Novel tobacco products such as EC and HTP, and their usage in combination with cigarettes, were not considered because of a lack of sufficient data over time that could serve as a basis for credible prediction^9^. In 2021 in Korea, the prevalences of EC and HTP use among adults aged >19 years were 2.5% and 3.8%, respectively, below the international average of 11%^41,42^. However, if the prevalence of EC or HTP use increases in Korea in the future, it could significantly influence the smoking initiation and cessation rates. Second, Korea SimSmoke depends on data limitations, the Markov model assumption as well as the parameter uncertainty^43^. For example, the SimSmoke model assumed an inflation rate of 3% from 2021. Therefore, if the inflation rate exceeds 3%, the cigarette price may be overestimated. In addition, there is potential for overestimation because we assumed a smoking initiation of zero for the TFG scenario whereas social supply is expected to be feasible^12^. Third, the SimSmoke population model did not take into account immigration^18^.

## CONCLUSIONS

To achieve the HP 2030 target, tobacco-control policies should be strengthened as soon as possible. In particular, to achieve the tobacco endgame by 2050, the Korean government needs to set clear goals and introduce innovative strategies such as the TFG and radical price increases. In addition, the timeframe of ending tobacco use by 2050 used in our research is longer than the international standard, which calls for achievement of the tobacco endgame within 20 years^44^. To realize the tobacco endgame before 2050, additional innovative control measures, such as nicotine content regulations and retail store restrictions^2^, which were not analyzed in this study, might need to be implemented in Korea.

## Data Availability

Data sharing is not applicable to this article as no new data were created.
