# Corrigendum: Modeling the future of tobacco control: Using SimSmoke to explore the feasibility of the tobacco endgame in Korea

**DOI:** 10.18332/tid/176286

**Published:** 2023-12-05

**Authors:** Hana Kim, Susan Park, Heewon Kang, Naeun Kang, David T. Levy, Sung-il Cho

**Affiliations:** 1 Department of Public Health Science, Graduate School of Public Health, Seoul National University, Seoul, Republic of Korea; 2 Institute of Health and Environment, Seoul National University, Seoul, Republic of Korea; 3 Lombardi Comprehensive Cancer Center, Georgetown University, Washington, United States

**Keywords:** SimSmoke, endgame, MPOWER, HP2030, modelling

Corrigendum on:

Modeling the future of tobacco control: Using SimSmoke to explore the feasibility of the tobacco endgame in Korea

By Hana Kim^1+^, Susan Park^2+^, Heewon Kang^2^, Naeun Kang^1^, David T. Levy^3^, Sung-il Cho^1,2^

Tobacco Induced Diseases, Volume 21, Issue November, Page 1-11, Publish date: 9 November 2023

DOI: https://doi.org/10.18332/tid/174127

In the corrected version of the article the co-first authorship of Hana Kim and Susan Park has been added. The mentioned change is corrected also online.

