# Supplementary material for: Modeling the future of tobacco control: Using SimSmoke to explore the feasibility of the tobacco endgame in Korea
Source: Tob Induc Dis. 2023 Nov 9;21:147. doi: 10.18332/tid/174127 (PMC10632939; doi:10.18332/tid/174127)
Supplement: Supplementary file 1 [file TID-21-147-s1.pdf]

Supplement 1. Estimating male (A) and female (B) smoking prevalence and confidence intervals from six intervention scenarios in 2050

(A)

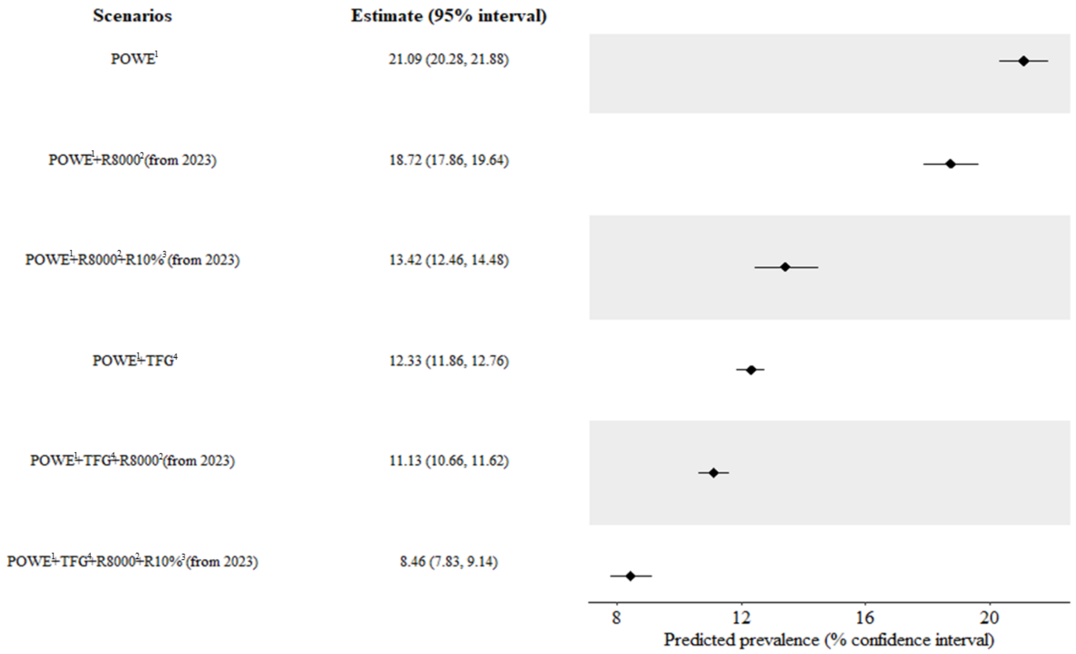

(B)

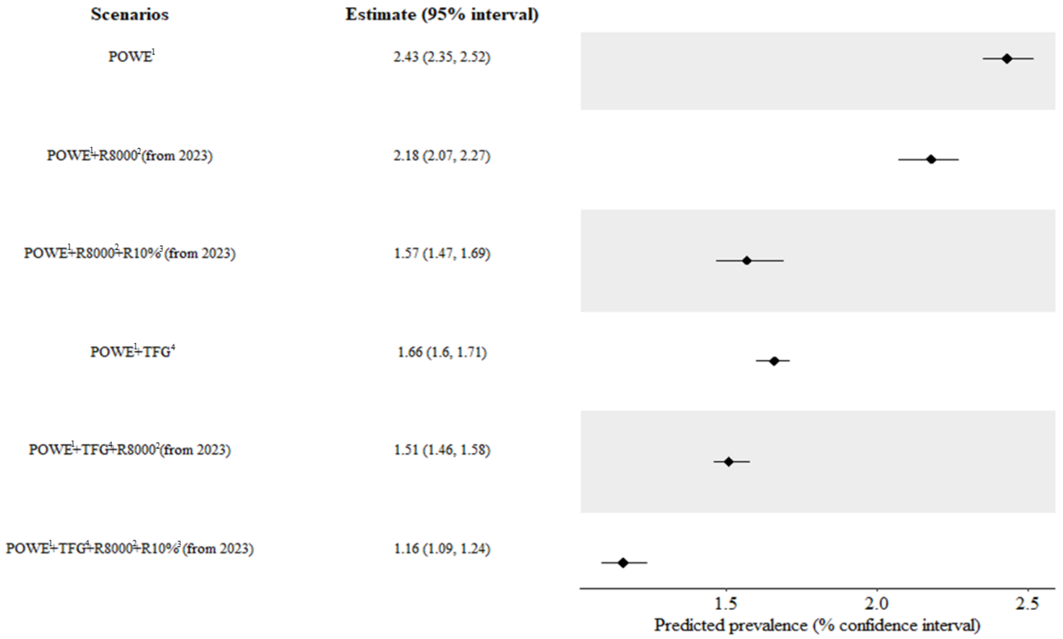

Apply each strengthened policy scenario from 2023

[1] **POWE**: P, protect people from tobacco smoke; O, offer help to quit tobacco smoking; W, warn of the dangers of tobacco; E, enforce bans on tobacco advertising, promotion, and sponsorship.

[2] **R8000:** Cigarette price increase to KRW 8000.

[3] **R10%:** Annual 10% cigarette price increase from KRW 8000.

[4] **TFG:** Tobacco-Free Generation. We assumed that persons born after 2003 would not start smoking.

Supplement 2. Smoking prevalence in SimSmoke, KNHANES, Social Statistics, and CHS by gender.

(A) Males

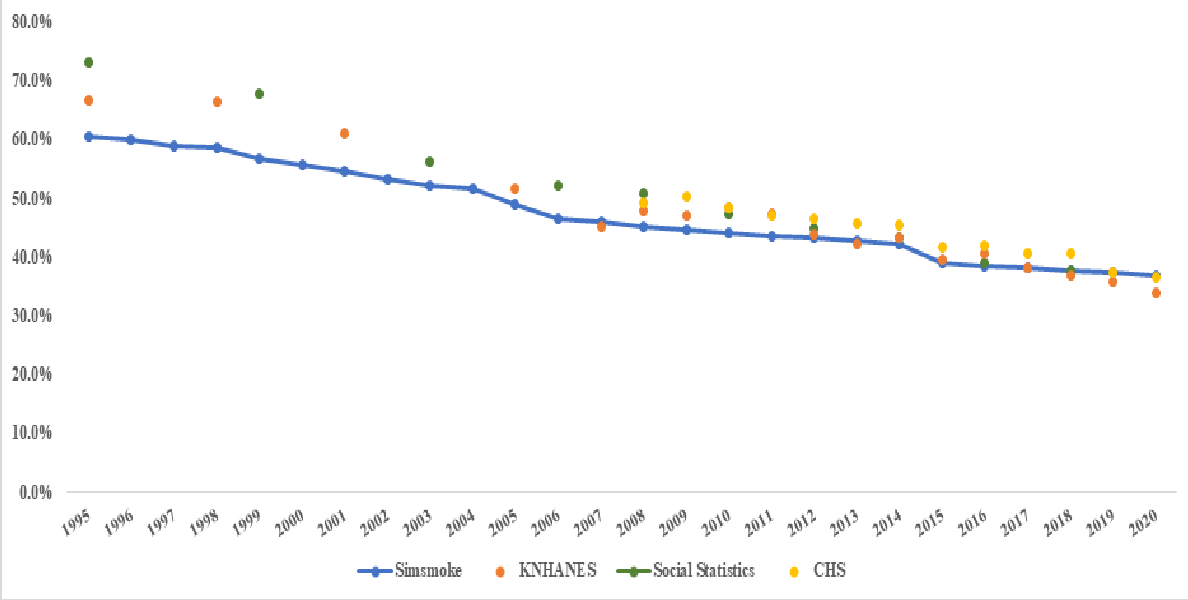

(B) Females*

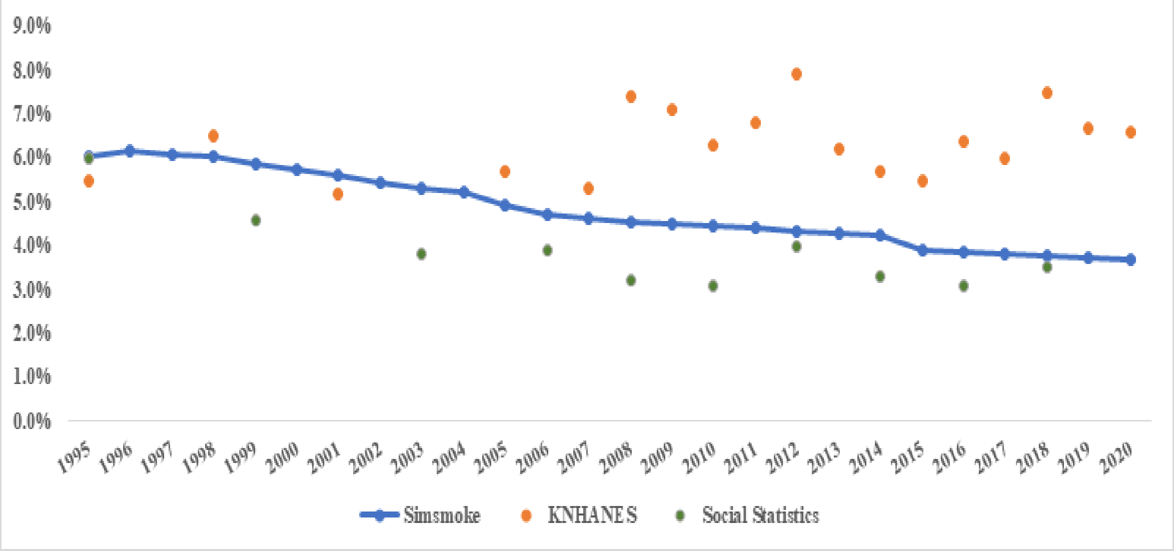

*The statistics of KNHANES, Social Statistics Survey, and CHS were based on publicly released data. CHS, Community Health Survey; KNHANES, Korea National Health and Nutrition Examination Surveys

Reference

1. Korea Disease Control and Prevention Agency. Korea National Health and Nutrition Examination Survey. Accessed March 02, 2022. Available from: https://knhanes.kdca.go.kr/knhanes/eng/index.do.
2. Statistics Korea. Korean Statistical Information Service. Accessed March 02 2022. Available from: https://kosis.kr/common/meta_onedepth.jsp?vwcd=MT_ZTITLE&listid=B_7. (Korean).
3. Korea Disease Control and Prevention Agency. Korea community health at a glance: Korea Community Health Survey (KCHS). Accessed March 02 2022. Available from: https://chs.kdca.go.kr/chs/stats/statsMain.do. (Korean, author's translation).

© 2023 Kim H. et al.
